# Hyphal editing of the conserved premature stop codon in *CHE1* is stimulated by oxidative stress in *Fusarium graminearum*

**DOI:** 10.1007/s44154-024-00174-w

**Published:** 2024-06-12

**Authors:** Jingwen Zou, Yanfei Du, Xiaoxing Xing, Panpan Huang, Zeyi Wang, Huiquan Liu, Qinhu Wang, JinRong Xu

**Affiliations:** 1 State Key Laboratory of Crop Stress Biology for Arid Areas and NWAFU-Purdue Joint Research Center, College of Plant Protection, Northwest A&F University, Yangling, 712100 Shaanxi China; 2 Dept. of Botany and Plant Pathology, Purdue University, West Lafayette, 47907 IN USA

**Keywords:** Editing during asexual reproduction, mRNA editing, Hyphal editing, ADAT-mediated mRNA editing, *CHE1*, Tad2 and Tad3

## Abstract

Although genome-wide A-to-I editing mediated by adenosine-deaminase-acting-on-tRNA (ADAT) occurs during sexual reproduction in the presence of stage-specific cofactors, RNA editing is not known to occur during vegetative growth in filamentous fungi. Here we identified 33 A-to-I RNA editing events in vegetative hyphae of *Fusarium graminearum* and functionally characterized one conserved hyphal-editing site. Similar to ADAT-mediated editing during sexual reproduction, majority of hyphal-editing sites are in coding sequences and nonsynonymous, and have strong preference for U at -1 position and hairpin loops. Editing at TA^437^G, one of the hyphal-specific editing sites, is a premature stop codon correction (PSC) event that enables *CHE1* gene to encode a full-length zinc fingertranscription factor. Manual annotations showed that this PSC site is conserved in *CHE1* orthologs from closely-related *Fusarium* species. Whereas the *che1* deletion and *CHE1*^TAA^ (G^438^ to A) mutants had no detectable phenotype, the *CHE1*^TGG^ (A^437^ to G) mutant was defective in hyphal growth, conidiation, sexual reproduction, and plant infection. However, the *CHE1*^TGG^ mutant was increased in tolerance against oxidative stress and editing of TA^437^G in *CHE1* was stimulated by H_2_O_2_ treatment in *F. graminearum*. These results indicate that fixation of the premature stop codon in *CHE1* has a fitness cost on normal hyphal growth and reproduction but provides a benefit to tolerance against oxidative stress. Taken together, A-to-I editing events, although rare (not genome-wide), occur during vegetative growth and editing in *CHE1* plays a role in response to oxidative stress in *F. graminearum* and likely in other fungal pathogens.

**Supplementary Information:**

The online version contains supplementary material available at 10.1007/s44154-024-00174-w.

## Introduction

Adenosines to inosine (A-to-I) editing mediated by ADAR (adenosine deaminase acting on mRNA) is the predominant form of mRNA editing in metazoans (Slotkin & Nishikura 2013; Eisenberg & Levanon 2018). Because of their similarity in base-pairing properties, inosines can be recognized as guanosines (G) during translation and reverse transcription. In humans, over three millions of A-to-I editing sites have been identified and defects in RNA editing is associated with health problems. Although lack of ADAR homologs, genome-wide A-to-I RNA editing is known to specifically occur during sexual reproduction in several filamentous ascomycetes, including *Neurospora crassa, Fusarium graminearum,* and *Sordaria macrospora* (Bian et al. 2018; Teichert et al. 2017). To date, over 40,000 sexual stage-specific editing sites have been identified in *F. graminearum*. The average editing level is less than 15%, which is similar to ADAR-mediated RNA editing in animals. However, unlike in animals, over 70% of the A-to-I editing sites are in the coding regions and majority of them result in amino acid changes, which significantly increase the proteome complexity in fungi. Studies with two conserved editing sites in the *CME5* and *CME11* genes have showed that nonsynonymous editing is adaptive and both edited and unedited transcripts of *CME11* are required for normal ascosporogenesis in *F. graminearum* (Xin et al. 2023).

ADAR-mediated RNA editing and A-to-I editing during sexual reproduction in fungi also differ significantly in the flanking nucleotide sequences and secondary RNA structures of the edited adenosines. Whereas ADARs with dsRNA-binding domains have no strong preference for the flanking sequences but preferentially edit adenosines in the stems (dsRNA regions) of mRNA. In filamentous ascomycetes, A-to-I editing strongly favors U at -1 position and majorities of editing sites are predicted to be in the hairpin loops. Because of the preference for U at -1, editing of stop codons often occur in fungi, including PSC (premature stop codon correction) editing and stop-loss editing (Liu et al. 2017). Recently, the *FgTAD2* and *FgTAD3* genes orthologous to yeast *TAD2* and *TAD3* ADAT (adenosine deaminase acting on tRNA) genes were found to be responsible for mediating stage-specific A-to-I editing in *F. graminearum* (Bian et al. 2024; Feng et al. 2023). These two ADATs form heterodimers to edit A34 in the anticodon loop of tRNA during vegetative growth but edit mRNA in the presence of stage-specific cofactors during sexual reproduction (Bian et al. 2024; Feng et al. 2023). Because A34 is preceded by U33 on the anticodon loop, the involvement of ADAT in mediating A-to-I editing in fungi explains the strong preference for U at -1 position and hairpin loops (Feng et al. 2022).

Although lack of genome-wide RNA editing in bacteria, 15 sites have been identified in *Escherichia coli*, in which the TadA ADAT protein itself (no cofactors involved) catalyzes rare A-to-I mRNA editing events (Bar-Yaacov et al. 2017). To determine whether ADAT-mediated mRNA editing can occur during vegetative growth in the absence of sexual-stage specific cofactors in fungi, in this study we analyzed RNA-seq data of vegetative hyphae and identified 33 A-to-I RNA editing sites in *F. graminearum*. Hyphal editing was found to have similar preferences for flanking nucleotide sequences and hairpin loops compared to RNA editing during sexual reproduction, and also tend to be in the coding region and nonsynonymous. One of the hyphal-specific RNA editing site is at the TA^437^G premature codon in the *CHE1* (conserved hyphal editing 1) gene. Whereas the *che1* and *CHE1*^TAA^ (non-correctable) mutants had no detectable phenotype, the *CHE1*^TGG^ mutant with the PSC site fixed in the genome were defective in growth and reproduction. However, editing of TA^437^G and tolerance against oxidative stress were increased in the *CHE1*^TGG^ mutant. Taken together, these results indicated that rare A-to-I RNA editing events do occur during vegetative growth and may play a role in responses to oxidative stress although genome-wide editing only occurs during sexual reproduction in the presence of stage-specific cofactors in *F. graminearum*.

## Results

### Identification and verification of rare A-to-I editing events in vegetative hyphae of *F. graminearum*

**Fig. 1:**
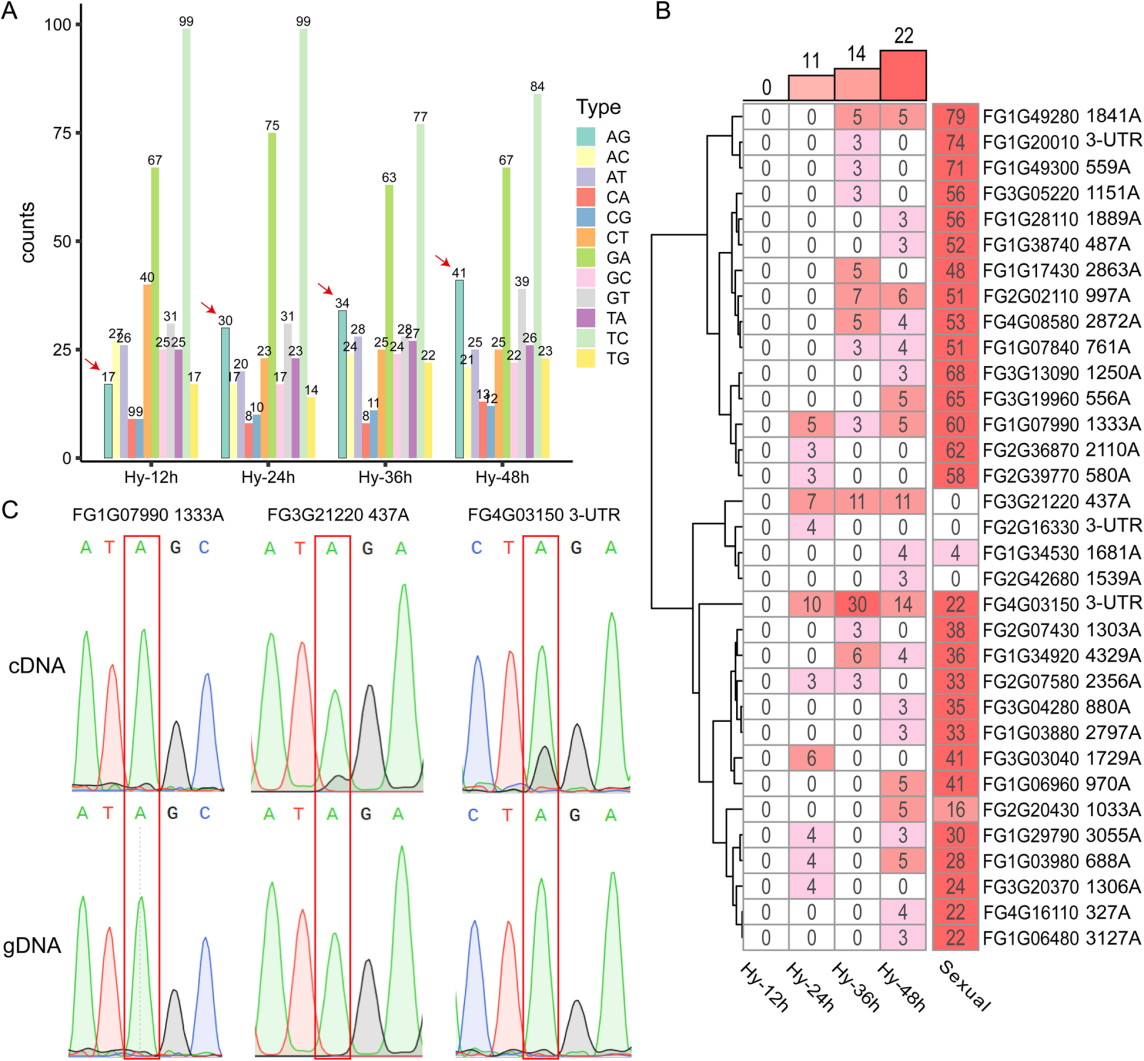
Identification of A-to-I editing events in vegetative hyphae. **A** The numbers of marked nucleotide variants in RNA-seq data of hyphae harvested from 12 to 48 h YEPD cultures. The AG variants (marked with arrows) increased from 12 to 48 h. **B** A-to-I editing sites in vegetative hyphae verified by manual examination. The values in the heatmap represent the editing level (%) of individual editing sites in hyphae (24, 36, and 48 h) and during sexual reproduction (sexual). Genomic locations and nucleotide changes in corresponding genes are listed on the right. **C** Fragments containing the editing sites of FG4G03150, FG3G21220, and FG1G07990 were amplified by RT-PCR from RNA isolated from 48 h hyphae (cDNA) or by PCR from genomic DNA (gDNA) and sequenced by Sanger sequencing. The dual peaks of A and G in cDNA samples indicate the occurrence of A-to-I editing at the marked sites

Whereas over 40,000 A-to-I mRNA editing sites have been identified in *F. graminearum* during sexual reproduction, there was no obvious enrichment of A-to-G variants in comparison with other nucleotide variants in RNA-seq data of hyphae freshly harvested from 12, 24, 36, and 48 h YEPD (yeast extract peptone dextrose) cultures (Lu et al. 2022). Nevertheless, we noticed that the number of AG variants increased from 12 to 48 h (Fig. 1A). Interestingly, such a trend was not observed for other nucleotide variants. After manual examination to remove the ones related to mapping errors, 11, 14, and 22 AG variants were identified in 24, 36, and 48 h hyphal samples, respectively (Fig. 1B). Among these 33 AG variants identified in 33 genes, nine were identified in more than one hyphal sample. Two had over 10% of G, with the highest being 30% at 24 h (Fig. 1B).

The only three AG variants common in 24, 36, and 48 h hyphae (Fig. 1B) were selected for verification. Fragments of the corresponding genes, FG4G03150, FG3G21220, and FG1G07990, were amplified by RT-PCR from RNA isolated from 48 h hyphae and sequenced by Sanger sequencing (Fig. 1C). For FG4G03150, approximately a third of its cDNA had A^2700^ in its 3’-UTR changed to G. For FG3G21220, over 10% of its cDNA had A^437^ in the coding region changed to G, resulting in the change of UA^437^G (stop codon) to UGG (W). For FG1G07990, the A^1333^ to G variant was observed at 3%. Because the read out of I is G in sequencing, these results indicated the occurrence of A-to-I editing at A^2490^, A^437^, and A^1333^ in FG4G03150, FG3G21220, and FG1G07990, respectively, in vegetative hyphae. Therefore, although genome-wide editing occurs during sexual reproduction with the involvement of stage-specific cofactors, rare A-to-I editing occurs to a limited number of genes during vegetative growth in *F. graminearum*.

### RNA editing in hyphae and perithecia has similar flanking sequence and structural preferences

**Fig. 2:**
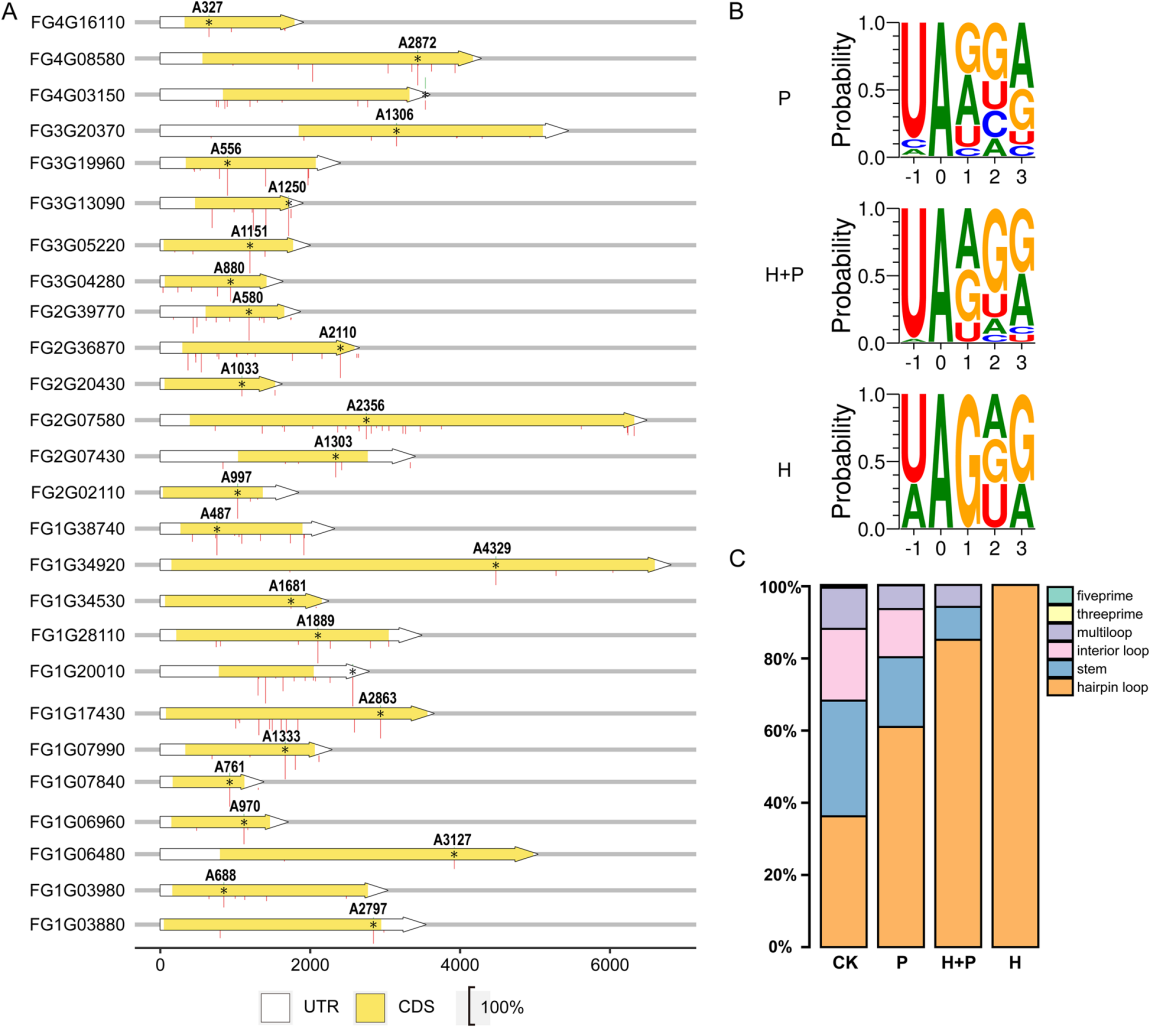
Similar preferences between RNA editing in hyphae and perithecia. **A** Diagrams of the 26 genes with one hyphal editing site (marked with * and green lines above) but two or more editing sites (marked with red lines below) during sexual reproduction. The height of green or red lines represents the editing level (with the scale bar of 100% shown below). UTR, 5’ or 3’ untranslated region; CDS, coding sequence. **B** Nucleotide sequences flanking the adenosines edited in both hyphae and perithecia (H + P), specifically in hyphae (H), or specifically in perithecia (P). **C** The frequency of editing events in predicted secondary structures of mRNA. All three hyphal-specific editing sites are on hairpin loops

When the 33 A-to-I hyphal editing sites were examined for their positions in the corresponding genes, only three of them are in the 3’-UTR regions (Table 1). The rest 30 (> 90%) are in the coding region, including 25 nonsynonymous editing and three UAG stop codon editing. Therefore, similar to editing during sexual reproduction, hyphal editing events also mainly occur in the coding region and are non-synonymous. In comparison with RNA editing during sexual reproduction, only three of the 33 hyphal editing sites were specifically edited during vegetative growth. The other 30 are also edited in perithecia, and all of them except the ones in FG1G34530 and FG4G03150 have higher editing levels during sexual reproduction (Fig. 1B). Among these 30 genes edited at both stages, 26 of them have two or more editing sites during sexual reproduction, with the site edited in hyphae having the highest editing level (Fig. 2A).

**Table 1:** A-to-I editing events detected in vegetative hyphae of *Fusarium graminearum*

Genomic Region	Strand	Gene Number ^a^ (Length of protein)	FgDB number (FGRRES-)	Position ^b^ of A to G variant	Amino acid change	Gene region	NonSyn	Unique to hyphae
Chr1:10,581,903	-	FG1G49280 (613)	10,343	1841	*614W^**d**^	Exon 5	Yes	
Chr1:10,587,157	+	FG1G49300 (249)	10,344	559	I187V	Exon 4	Yes	
Chr1:1,397,755	-	FG1G06480 (1393)	00434	3127	K1043E	Exon 5	Yes	
Chr1:1,488,191	-	FG1G06960 (438)	00467	970	R324G	Exon 2	Yes	
Chr1:1,645,633	-	FG1G07840 (318)	00526	761	Y254C	Exon 2	Yes	
Chr1:1,688,956	+	FG1G07990 (575)	00534	1333	S445G	Exon 1	Yes	
Chr1:3,778,179	-	FG1G17430 (1162)	01156_M	2863	K955E	Exon 3	Yes	
Chr1:4,330,242	+	FG1G20010 (421)	01309	+ 526 ^**c**^	-	3'UTR	-	
Chr1:6,111,247	-	FG1G28110 (944)	01854	1889	Y630C	Exon 2	Yes	
Chr1:6,475,796	-	FG1G29790 (1781)	15914_M	3055	K1019E	Exon 2	Yes	
Chr1:7,407,178	+	FG1G34530 (696)	02287	1681	K561E	Exon 1	Yes	
Chr1:7,525,039	-	FG1G34920 (2149)	02324	4329	L1443L	Exon 5	No	
Chr1:820,767	-	FG1G03880 (966)	00261	2797	N933D	Exon 1	Yes	
Chr1:8,369,095	-	FG1G38740 (542)	20,078	487	K163E	Exon 4	Yes	
Chr1:847,272	+	FG1G03980 (869)	00270	688	K230E	Exon 1	Yes	
Chr2:1,554,457	+	FG2G07430 (576)	08476	1303	K435E	Exon 2	Yes	
Chr2:1,580,468	+	FG2G07580 (1976)	08487_M	2356	N786D	Exon 3	Yes	
Chr2:3,464,922	+	FG2G16330 (349)	02812	+ 310 ^**c**^	-	3'UTR	-	Yes
Chr2:4,235,855	-	FG2G20430 (491)	12,507	1033A > G	K345E	Exon 2	Yes	
Chr2:459,140	-	FG2G02110 (443)	17097_M	997	R333G	Exon 2	Yes	
Chr2:7,536,494	-	FG2G36870 (765)	04295_M	2110	K704E	Exon 8	Yes	
Chr2:8,190,623	+	FG2G39770 (349)	04482	580	S194G	Exon 2	Yes	
Chr2:8,835,759	+	FG2G42680 (626)	14,016	1539	L513L	Exon 4	No	Yes
Chr3:1,027,059	-	FG3G05220 (575)	05060	1151	Y384C	Exon 1	Yes	
Chr3:2,754,956	+	FG3G13090 (416)	05580	1250	*417W^**d**^	Exon 2	Yes	
Chr3:4,124,823	+	FG3G19960 (577)	06045_M	556	K186E	Exon 2	Yes	
Chr3:4,216,246	+	FG3G20370 (1085)	06071	1306	K436E	Exon 2	Yes	
Chr3:4,382,711	-	FG3G21220 (145/310) ^**e**^	12,869	437	*146W^**d**^	Exon 2	Yes	Yes
Chr3:636,401	+	FG3G03040 (874)	04916	1729	K577E	Exon 2	Yes	
Chr3:851,069	-	FG3G04280 (452)	04996	880	R294G	Exon 3	Yes	
Chr4:1,887,526	+	FG4G08580 (1202)	06970	2872	K958E	Exon 3	Yes	
Chr4:3,525,801	+	FG4G16110 (488)	16,831	327	I109M	Exon 2	Yes	
Chr4:652,840	+	FG4G03150 (829)	06630	+ 210 ^**c**^	-	3'UTR	-	

^**a**^Gene numbers based on the latest annotation. In the bracket are the numbers of amino acid residues encoded by each gene

^**b**^Unless specified, it is the A-to-G change site in the ORF of predicted genes (with A in ATG as 1)

^c^The symbol ' + " denote the occurrence of editing in the 3'-UTR, at the number of base pairs downstream from the stop codon of the predicted ORF

^**d**^Editing of stop codon UAG

^**e**^Unedited and edited transcripts encode 145 and 310 amino acid residues, respectively

Similar to RNA editing during sexual reproduction, there is a strong preference for U at -1 position for hyphal editing sites (Fig. 2B), which is not surprising because only three of those hyphal editing sites are unique to vegetative hyphae and they also strongly favor U at the -1 position. Furthermore, hyphal editing also tends to prefer G/A at + 1 and + 3 positions (Fig. 2B). Editing sites in hyphae also had a stronger preference for hairpin loops (over 80%), with all three hyphal-specific sites in hairpin loops (Fig. 2C). Based on these observations, we conclude that RNA editing in vegetative hyphae, although rare, has similar preference for flanking sequences, especially for the nucleotide at -1 position and hairpin loops in secondary structures with genome-wide editing during sexual reproduction, indicating the involvement of the same ADAT-mediated editing mechanism.

### *CHE1* and its PSC editing site are conserved in closely-related *Fusarium* species

**Fig. 3:**
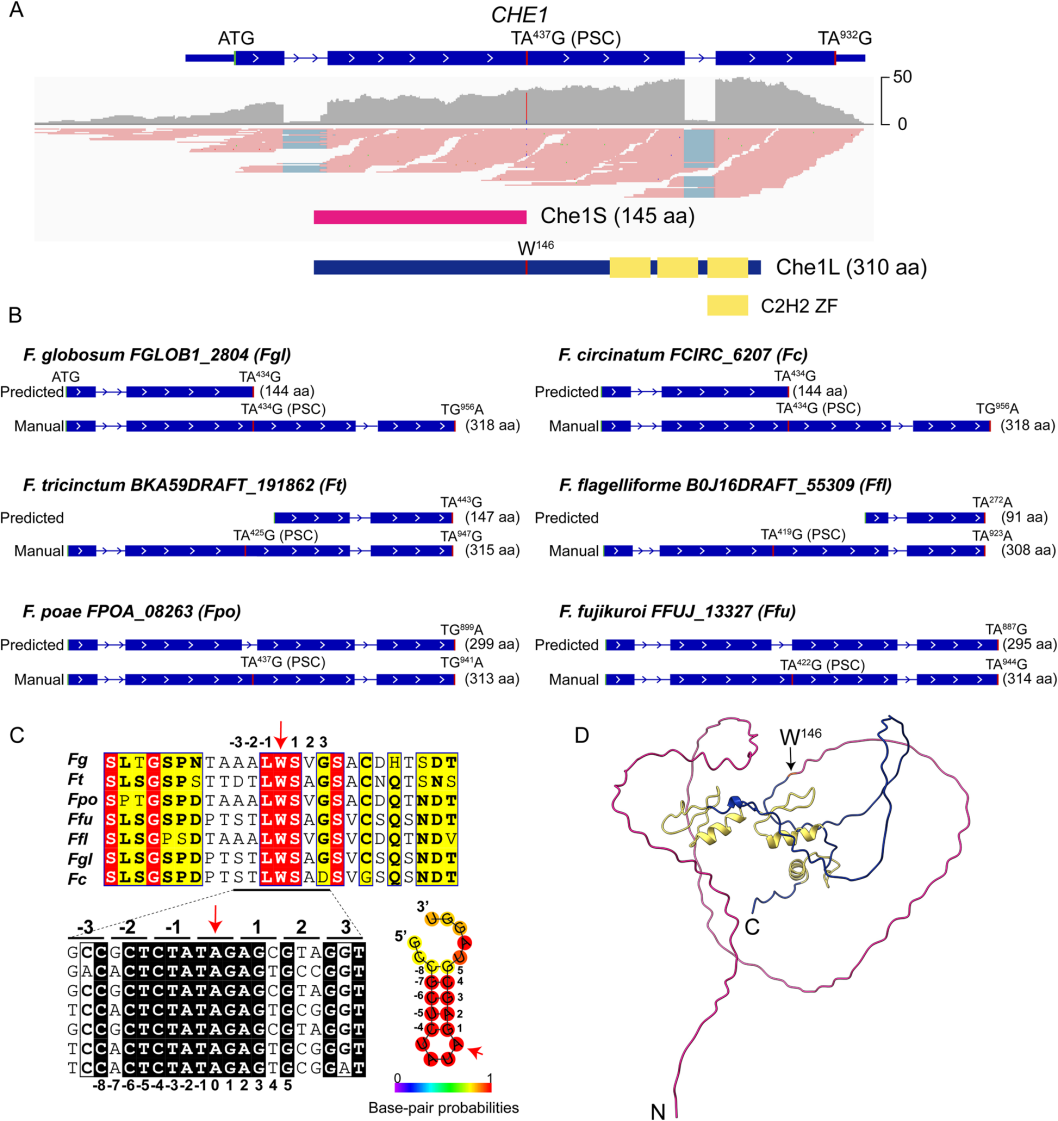
The conserved PSC site in *CHE1* orthologs from closely related *Fusarium* species. **A** A diagram of the *CHE1* gene showing the PSC site and domain structures of Che1 proteins. Yellow boxes, C2H2 ZF domains. **B** Three types of annotation problems caused by the conserved PSC site in *CHE1* orthologs. Like in *F. graminearum*, the PSC site is predicted to be canonical stop codon in *F. globosum* (*Fgl*) and *F. circinatum* (*Fc*). In *F. tricinctum* (*Ft*) and *F. flagelliforme* (*Ffl*), the first methionine codon downstream from the PSC site is predicted to be the start codon. In *F. poae* (*Fpo*) and *F. fujikuroi* (*Ffu*), a small intron is erroneously introduced by automated annotation to splice out the PSC site. **C** Sequence alignment of amino acid residues and nucleotides (corresponding to -3 to + 3 aa) flanking the PSC editing site in Che1 and its orthologs from the same *Fusarium* species. Arrows point to the editing site in *CHE1*. Identical and similar amino acid residues are shaded in red and yellow, respectively. Identical nucleotides are shaded in black. On the right is the predicted location of A^437^ in hairpin loops based on the nucleotide sequences of -3 to + 3 amino acid residues in *CHE1* and its orthologs. **D** Protein structure of Che1L predicted by AlphaFold2. Three C2H2 ZF domains are in gold

Among the three hyphal stop editing sites (TAG), two are at the canonical stop codons of FG1G49280 and FG3G13090, resulting in C-terminal extension of 28 aa and 11 aa, respectively. However, editing of A^437^ occurs at TA^437^G, a premature stop codon in the middle of FG3G21220 (FGRRES_12869, named *CHE1* for conserved hyphal editing 1), is a PSC editing event occurred during vegetative growth. Without editing of A^437^, *CHE1* transcripts encode a 145-aa protein (Chs1S) that has no known motif, which is the predicted protein encoded by FGRRES_12869 based on automated annotation (Table 1). However, the full-length 310 aa protein (Che1L) encoded by *CHE1* transcripts after TA^437^G editing contains three C2H2 zinc finger (ZF) domains in the C-terminal region (Fig. 3A).

Interestingly, this PSC site is well-conserved in *CHE1* homologs from closely related *Fusarium* species, which causes annotation problems (Fig. 3B). Whereas the *CHE1* homolog in many species is predicted to encode a Che1S-like protein, a small intron was erroneously introduced by automated annotation to splice out this conserved PSC site in other species (Fig. 3B). In a few species such as *F. tricinctum* and *F. flagelliforme*, the methionine codon downstream from the PSC site is predicted to be the start codon (Fig. 3B). However, there is no conserved methionine codons downstream from the PSC site in *CHE1* homologs. In *F. graminearum*, if A^711^TG, the first methionine codon downstream from TA^437^G, acts as the start codon, the resulting 63-aa protein contains only parts of C2H2 ZF domains.

Sequence alignment with manually annotated Che1 orthologs from closely related Fusarium species showed that the 1–145 aa region of Che1 shares approximately 52.8% identity in amino acid sequences, which is less than 62.6% identity in the 146–437 aa region containing the three C2H2 zinc finger domains (Fig. S1). Interestingly, based on the conserved amino acid residues flanking W136 and their corresponding nucleotide sequences, A^437^ is predicted to be on a hairpin loop in these *CHE1* homologs (Fig. 3C). When predicted by AlphaFold2, the three C2H2 ZF domains of Che1 appear to be in the center (Fig. 3D). The long N-terminal region (1–220 aa) with no predictable structures is on the surface of Che1 protein.

### Fixation of the PSC site of *CHE1* in the genome has a detrimental effect on hyphal growth

**Fig. 4:**
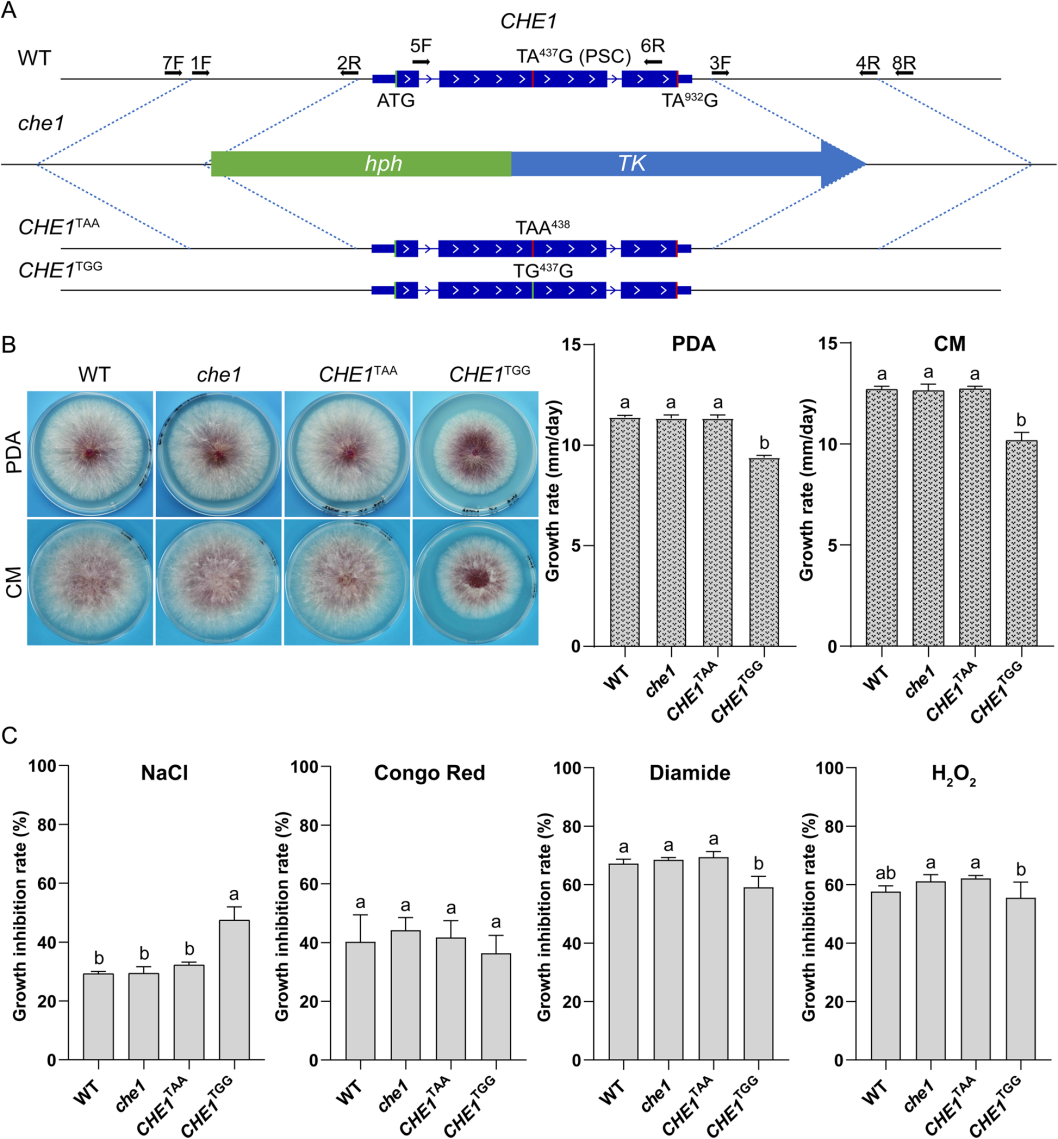
Phenotypes of the *che1*, *CHE1*^TAA^, and *CHE1*^TGG^ mutants. **A** A schematic draw showing the *CHE1* gene and generation of the *che1* deletion, *CHE1*^TAA^, and *CHE1*^TGG^ mutants. The direction of *CHE1* and primers used to generate mutants were marked with arrows. *hph*, hygromycin phosphotransferase gene; *tk*, thymidine kinase gene. **B** Three-day-old PDA and CM cultures of PH-1 (WT) and the *che1*, *CHE1*^TAA^, and *CHE1*^TGG^ mutants were examined for colony morphology (left) and measured for growth rate (right). **C** The diameter of colonies formed by the labelled strains on regular CM and CM with the addition of 0.7 M NaCl, 300 μg/ml Congo Red, 0.05% H_2_O_2_, or 2 mM diamide was measured after incubation for three days. For each treatment, the inhibition rate = (colony diameter with the treatment/colony diameter on regular CM) × 100%. For B) and c), mean and standard deviations are calculated with data from three biological replicates. Different letters mark significant differences (*p* = 0.05) based on one-way ANOVA analysis

To determine the role of *CHE1* and its PSC editing, we first generated the *CHE1* gene replacement construct with the *hph-tk* cassette and transform it into the wild-type strain PH-1 (Fig. 4A). Transformants resistant to hygromycin but sensitive to floxuridine were isolated and verified for the deletion of *CHE1* (Fig. S2). The *che1* deletion mutant had normal growth rate and colony morphology (Fig. 4B). We then used the overlapping PCR approach to introduce the TA^437^G^438^ to TG^437^G (fixed in the genome) or TAA^438^ (un-correctable) mutation into *CHE1*. The resulting mutant alleles were verified by sequencing and transformed into the *che1* mutant. Because the *tk* gene confers sensitivity to nucleoside analog floxuridine (Qi et al. 2024), transformants sensitive to hygromycin but resistant against floxuridine were isolated and verified by PCR for having the transforming mutant alleles integrated at the endogenous *CHE1* locus to replace the *hph-tk* cassette (Fig. S3).

The *CHE1*^TAA^ mutant, like the *che1* mutant, had no growth defects. However, the *CHE1*^TGG^ mutant was reduced in growth rate although it had normal colony morphology (Fig. 4B). On both PDA or CM plates, the growth rate was reduced approximately 20% in the *CHE1*^TGG^ transformant compared to PH-1 or *che1* mutant (Fig. 4B). When assayed for growth inhibition by different stressors, the *che1* and *CHE1*^TAA^ mutants had no obvious defects in stress responses. However, the *CHE1*^TGG^ mutant was more tolerant to oxidative stress induced by diamide or H_2_O_2_ treatment although it was more sensitive to osmotic stress compared to PH-1 (Fig. 4C). These results indicate that fixation of the premature stop codon in *CHE1* has a fitness cost on normal hyphal growth but provides a benefit to tolerance against oxidative stress in *F. graminearum*.

### The *CHE1*^TGG^ transformant is defective in asexual and sexual reproduction

**Fig. 5:**
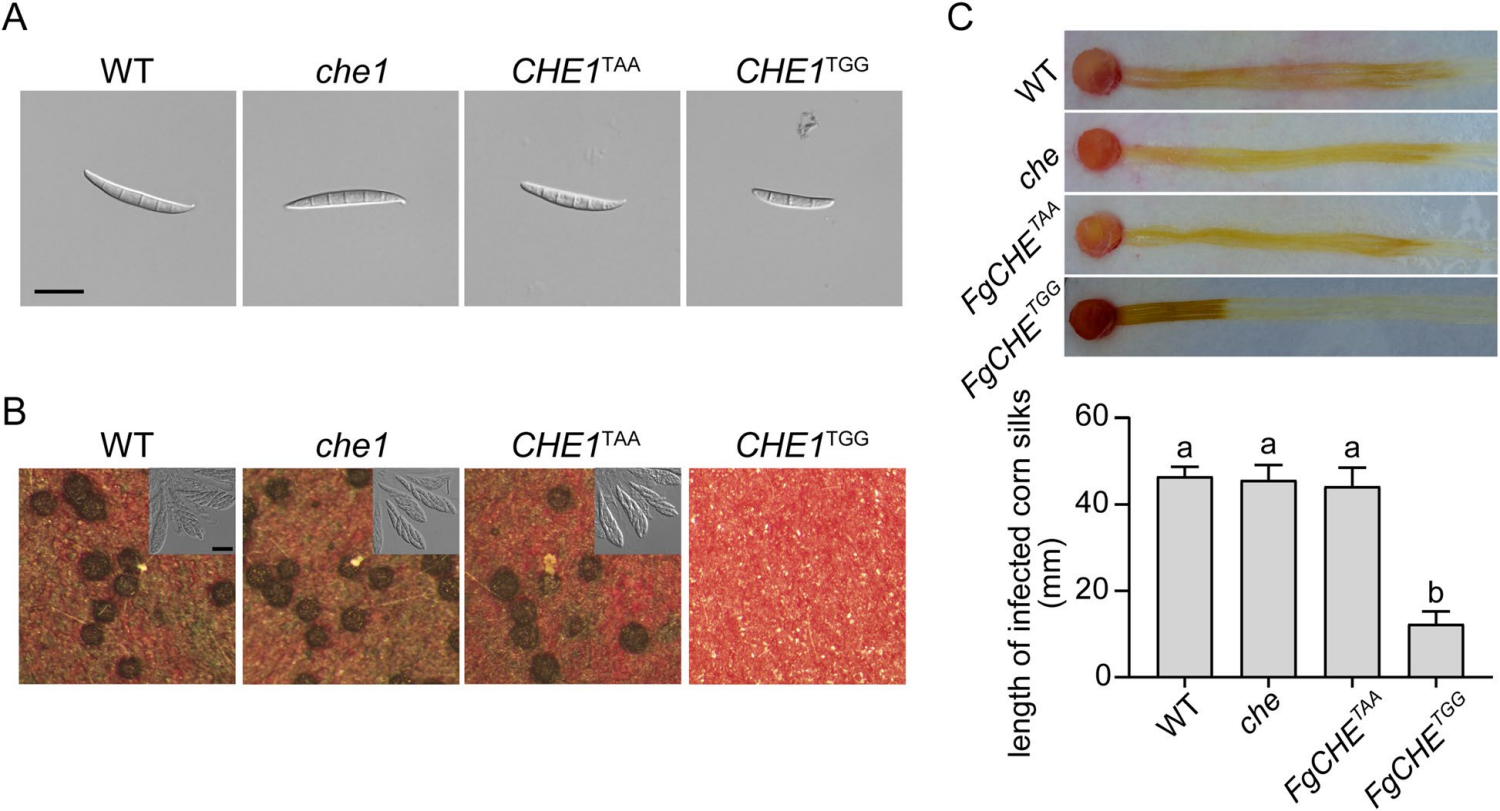
Defects of the *CHE1*^TGG^ mutant in sexual and asexual reproduction. **A** Conidiation in 5-day-old CMC cultures of PH-1 (WT) and the *che1*, *CHE1*^TAA^, and *CHE1*^TGG^ mutants. **B** Mating cultures of the labelled strains were examined for perithecium development (upper) and ascus/ascospore formation at 14 days post-fertilization (pdf). **C** Corn silks inoculated with the labelled strains were examined for disease symptoms and measured for the length of discolored lesions at 5 dpi. Bar = 20 µm. For A) and D), mean and standard deviations are calculated with data from three biological replicates. Different letters mark significant differences (*p* = 0.05) based on one-way ANOVA analysis

When assayed for conidiation in 5-day-old CMC cultures, the *che1* and *CHE1*^TAA^ mutants were similar to the wild type and produced conidia with normal morphology (Fig. 5A). However, the *CHE1*^TGG^ mutant was significantly reduced in conidiation. Rare conidia produced by the *CHE1*^TGG^ mutant also tended to be shorter and have fewer septa than those of the wild type (Fig. 5A), indicating that *CHE1* is dispensable for conidiation but the TGG mutation in *CHE1* has a detrimental effect on conidiogenesis.

In mating cultures on carrot agar plates, abundant perithecia with normal asci and ascospores were formed by the wild type and the *che1* and *CHE1*^TAA^ mutants at 14 days post-fertilization (dpf). Under the same conditions, the *CHE1*^TGG^ mutant was sterile and failed to produce perithecia on mating plates (Fig. 5B). Even after incubation for four weeks, no perithecia were observed in the *CHE1*^TGG^ mutant. In infection assays with corn silks, the *che1* and *CHE1*^TAA^ mutants were normal but the *CHE1*^TGG^ mutant was significantly reduced in virulence (Fig. 5C). These results indicate that deletion of *CHE1* or the TAA mutation has no effect but fixation of the PSC site in *CHE1* has an inhibitory effect on sexual reproduction and pathogenesis in *F. graminearum*.

### Editing of the PSC site in *CHE1* is increased by H_2_O_2_ treatment

**Fig. 6:**
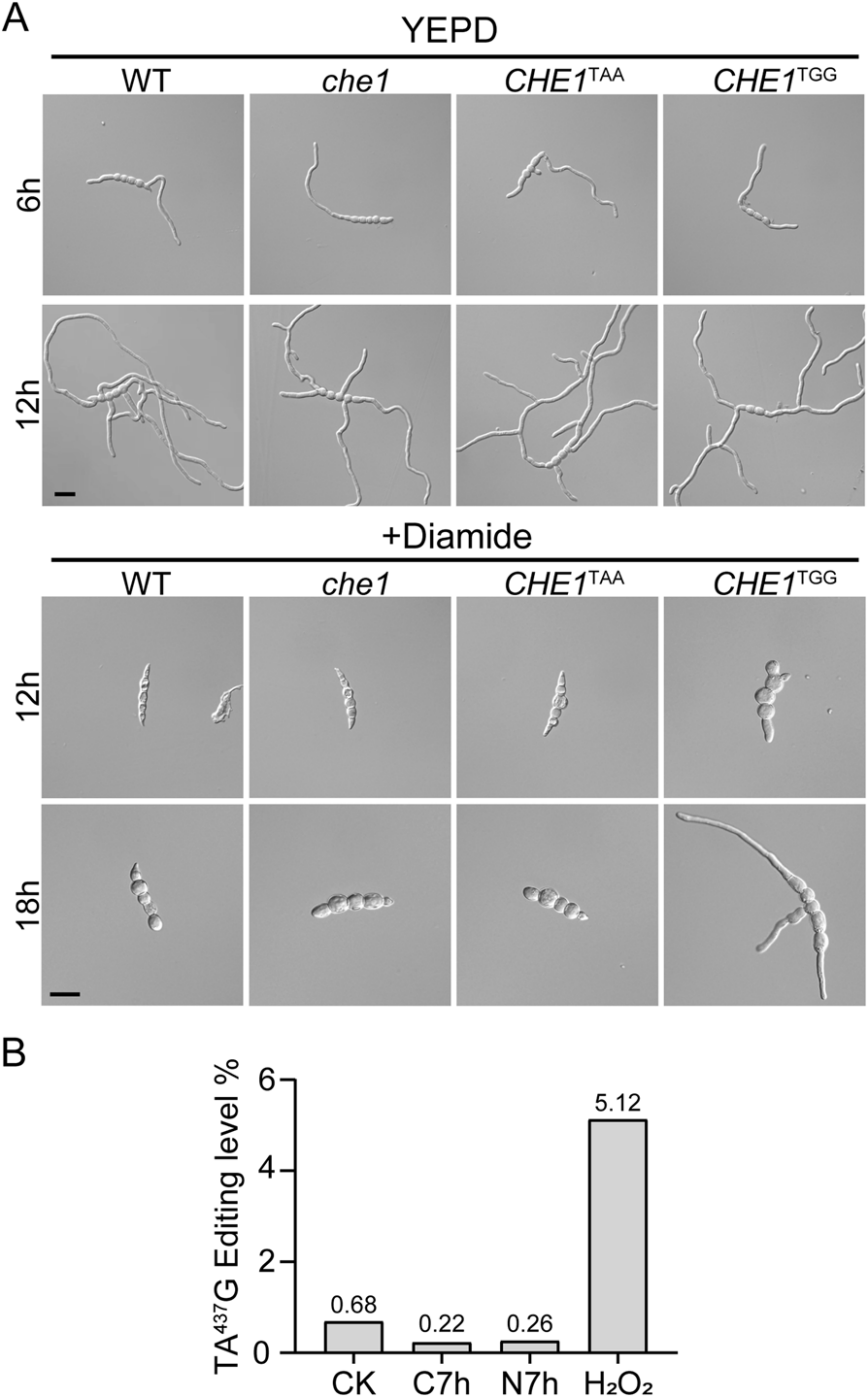
Editing of the PSC site in *CHE1* is related to oxidative stress. **A** Germination and germ tube growth were examined after incubation of conidia of the labelled strains in regular YEPD for 6 or 12 h and YEPD with 2 mM diamide for 12 or 18 h. Bar = 20 µm. **B** Assay for editing levels of the PSC site in *CHE1* in hyphae of PH-1 starved for nitrogen (N7h) or carbon (C7h) sources for 7 h or treated with 0.05% H_2_O_2_ for 2 h by RT-PCR and wide-seq analysis. CK, the untreated hyphae cultured in YEPG for 7 h

To verify the effect of TGG mutation in *CHE1* on tolerance against oxidative stress, we assayed conidium germination in the presence of 0.05% mM H_2_O_2_. After incubation in regular YEPD medium for 6 or 12 h, conidium germination and initial germ tube growth were similar between the wild type and the *che1*, *CHE1*^TAA^, and *CHE1*^TGG^ mutants (Fig. 6A). However, in the presence of 2 mM diamide, conidium germination was not observed in the wild type and the *che1* and *CHE1*^TAA^ mutants after incubation for 12 or 18 h. Under the same conditions, germination and germ tube growth were observed in the *CHE1*^TGG^ mutant (Fig. 6A), confirming that fixation of the PSC site in *CHE1* confers tolerance to oxidative stress.

To determine the effect of oxidative stress on editing of the PTC in *CHE1*, we treated freshly harvested hyphae with 0.05% H_2_O_2_. In comparison with untreated control (0.68%), the editing level at A^437^ (5.12%) was significantly increased by H_2_O_2_ treatment (Fig. 6B). We also tested culture conditions with no carbon or nitrogen sources. Starvation for nitrogen or carbon had no stimulatory effects on editing in *CHE1* (Fig. 6B). These results indicated that editing of the PSC site in *CHE1* is stimulated by oxidative stress and the full-length Che1 protein may play a role in regulating responses to oxidative stress in *F. graminearum*.

## Discussion

Earlier studies have identified over 40,000 A-to-I RNA editing sites that occurs in majority of genes expressed during sexual reproduction in *F. graminearum*. However, the enrichment of AG variants was not observed in hyphae in these studies, indicating the lack of genome-wide A-to-I RNA editing during vegetative growth. In this study, after a careful examination of published RNA-seq data (Lu et al. 2022), 33 A-to-I editing sites were identified in 33 genes expressed in hyphae*,* with three of them being confirmed by RT-PCR and sequencing. On average, the editing level of these hyphal editing events is less than 5.5%. Therefore, editing events in vegetative hyphae are rare (not genome-wide without the sexual-specific cofactors) and have relatively low editing levels in *F. graminearum*, making it impossible to identify hyphal editing by simply searching for AG variant enrichment (Lu et al. 2022; Liu et al. 2016).

Because 30 of the 33 hyphal editing sites are also edited in perithecia, it is not surprising that RNA editing during vegetative growth and sexual reproduction has similar sequence and structure preferences*.* All three sites specifically edited in vegetative hyphae, including the PSC site in *CHE1*, are on hairpin loops and strongly favors U at -1 position, which is similar to A34 on the anticodon loop edited by ADATs (Torres et al. 2014). Therefore, RNA editing in hyphae, similar to RNA editing during sexual reproduction, is likely mediated by ADATs in *F. graminearum*. In *E. coli*, all 15 mRNA editing sites have the same sequence and structure preferences with A34 in tRNA^Arg^ (Bar-Yaacov et al. 2017). It is likely that the FgTad2-FgTad3 ADAT heterodimer, besides being responsible for tRNA editing, can selectively and inefficiently edit certain mRNA during vegetative growth, resulting rare hyphal editing events with low editing levels. During sexual reproduction, genome-wide RNA editing occurs in the presence of stage-specific cofactors.

Regarding the functions of hyphal editing, although only 33 editing sites were identified in this study, 30 of them are in the CDS and majority of them cause changes in amino acid sequences, including three stop codon editing events, which is similar to features of A-to-I mRNA editing during sexual reproduction. Therefore, we expect that hyphal editing will have similar effects with sexual editing on increasing proteosome complexity and providing adaptive advantages (Xin et al. 2023; Qi et al. 2024; Lu et al. 2022). For the PSC editing site in *CHE1*, the *CHE1*^TGG^ mutant was blocked in sexual reproduction and reduced in growth rate, conidiation, and virulence, indicating that fixation of this hyphal editing site in the genome has detrimental effects on growth and reproduction. However, editing of TA^437^G to TGG in *CHE1* appears to be beneficial to tolerance against oxidative stress. The *CHE1*^TGG^ mutant was increased in tolerance against oxidative stress and editing of TA^437^G was stimulated by H_2_O_2_ treatment. For other hyphal editing sites, some of them may also have functions in *F. graminearum*. In bacterial pathogen *Xanthomonas oryzae*, mRNA editing is rare but the editing event in a ferric siderophore receptor gene is important for normal virulence (Nie et al. 2020).

With three conserved zinc finger domains at the C-terminal region, the full-length Che1L protein likely functions as a transcription factor belonging to the C2H2 zinc finger family (Fedotova et al. 2017). However, due to low abundance of *CHE1* transcripts and 9–10% editing level at A^437^ in hyphae, Che1L protein is expressed at a relatively low level. Majority of *CHE1* transcripts encode the 145-aa Che1S protein, which lacks any structural feature or domain. Whereas the *che1* mutant was normal, the *CHE1*^TGG^ mutant was reduced in growth rate but increased tolerance against oxidative stress, Che1L is likely involved in negatively regulating subsets of genes important for normal hyphal growth and positively regulating genes required for responses to oxidative stress but detrimental to hyphal growth. In the *CHE1*^TGG^ mutant, Che1L is overexpressed compared to the wild type and Che1S expression is eliminated. The *CHE1*^TGG^ mutant was blocked in sexual reproduction and significantly reduced in conidiation. These defects may be related to the repression of genes important for conidiogenesis and protoperithecium development by Che1L overexpression in the *CHE1*^TGG^ mutant. Although *CHE1* homologs are conserved in closely related *Fusarium* species and likely have the same functions, none of them were correctly annotated by automated gene prediction. Due to the conserved PSC site in the middle of ORF, they were often predicted to encode only the N-terminal or C-terminal region and have to be manually corrected by converting TAG to TGG. Unfortunately, the N-terminal region upstream from the PSC site is not well conserved in non-*Fusarium* species and the C-terminal region contains three C2H2 zinc finger domains, which are present in members of a large transcription factor family. Therefore, it is very challenging, if not impossible, to systematically analyze the distribution of *CHE1* homologs in fungi, particularly fungi outside Sordariomycetes. Nevertheless, a careful examination showed that the budding and fission yeasts lack *CHE1*-like genes.

In summary, in this study we showed that A-to-I editing events, although rare (not genome-wide), do occur during vegetative growth in *F. graminearum*, which has not been reported in any other fungi. Unlike genome-wide RNA editing during sexual reproduction with sexual stage-specific cofactors, limited ADAT-mediated RNA editing may occur inefficiently in vegetative hyphae, which is similar to RNA editing in *E. coli*. Nevertheless, we showed that editing of the PSC site in *CHE1* provides benefits for increased tolerance against oxidative stress although has detrimental effects on growth, reproduction, and pathogenesis. It is likely that the expression of subsets of genes requires the full-length functional Che1 protein and some of these genes regulated by Che1 may be involved in normal hyphal growth and responses to various biotic and abiotic stresses. Therefore, further studies are necessary to identify and characterize other hyphal editing events under different biotic and abiotic stress conditions as well as molecular mechanisms regulating the expression and editing of *CHE1* in *F. graminearum*.

## Materials and methods

### Strains and culture conditions

The wild-type strain PH-1 of *F. graminearum* and mutants generated in this study were routinely maintained on potato dextrose agar (PDA) at 25 °C. Conidiation in 5-day-old carboxymethylcellulose (CMC) cultures, growth rate on 3-day-old complete medium (CM) cultures, and infection of corn silks were assayed as described (Hou et al. 2002; Wang et al. 2011a; Ren et al. 2022). For testing sensitivities against various stresses, the final concentration of 0.7 M NaCl, 300 μg/ml Congo Red, 200 μg/ml CFW, 0.05% H_2_O_2_, or 2 mM diamide was added to CM or YEPD (yeast extract peptone dextrose) (Duan et al. 2024; Wang et al. 2011b). Carrot agar cultures were used for assaying defects in sexual reproduction as described (Ding et al. 2023). For transformant selection in polyethylene glycol (PEG)-mediated transformation with protoplasts, hygromycin B (MDbio, China) and 5-fluoro-2’-deoxyuridine (floxuridine) (MCE, USA) were added to the final concentration at 200 μg/ml and 25 μg/ml, respectively, to the top agar (Qi et al. 2024; Huang et al. 2024).

### Identification and verification of A-to-I editing sites in vegetative hyphae

For identifying hyphal editing sites, RNA-seq data were mapped to the reference genome of PH-1_YL (Lu et al. 2022) with Hisat2 v2.2.1 (Kim et al. 2015). All the aligned bam files were deduplicated by Picard v2.18.7 MarkDuplicates (broadinstitute.github.io/picard/). The strand-specific RNA-seq bam files were then split into positive and negative strands with Bamtools v2.5.2 (Barnett et al. 2011). AG variants with coverage ≥ 10 and editing ≥ 3% were identified with REDItoolDnaRNA.py in REDItools v1.2 (Picardi & Pesole 2013). The AG variants identified by these analyses were manually inspected on the IGV (Thorvaldsdottir et al. 2013) of corresponding genes.

To verify the editing sites identified in FG4G03150, FG3G21220 (*CHE1*), and FG1G07990, RNA was isolated from 48 h YEPD cultures of PH-1 with the Eastep Super Total RNA Extraction Kit (Promega Shanghai, China). Corresponding cDNA fragments were amplified by RT-PCR and sequenced by Sanger sequencing. Same regions of these genes were amplified from genomic DNA and sequenced for comparison. Traces from Sanger sequencing were visualized using SnapGene Viewer 4.3 (www.snapgene.com/snapgene-viewer/).

### Analysis of hyphal editing features and corresponding genes

The flanking nucleotides of edited adenosines were visualized with Weblogo 3 (Crooks et al. 2004). Secondary structures of RNA were predicted with RNAfold in Viennarna v2.4.18 (Lorenz et al. 2011) and analyzed with forgi v2.0.3 (Thiel et al. 2019) for favored secondary structures for editing. Genes with hyphal editing sites were identified and annotated with Transvar v2.5.9 (Zhou et al. 2015). For gene expression, RNA-seq data were mapped to the reference genome with Hisat2 v2.2.1. The number of fragments per gene was counted by featureCounts v2.0.6 (Liao et al. 2014) and converted to TPM (Transcripts Per Million) with R 4.1.2 (www.r-project.org).

### Assay *CHE1* editing in hyphae cultured under different conditions

Hyphae of PH-1 were harvested from 200 mL YEPD cultures after incubation for 24 h by filtration, washed once with sterile distilled water, and divided into four aliquots for further incubations under different conditions. For the control, hyphae were incubated in 100 mL fresh YEPD for 7 h. For carbon or nitrogen starvation, hyphae were incubated in 100 mL minimal medium (MM: 20 g glucose, 1 g NH_4_NO_4_, 1 g KH_2_PO_4_, 0.5 g KCl, 0.5 g MgSO_4_, and 0.3 g FeSO_4_ per liter) without the carbon (-glucose) or nitrogen (-NH_4_NO_4_) source for 7 h. For oxidate stress, hyphae were incubated in 100 mL YEPD with 0.05% H_2_O_2_ for 2 h. For all four samples, RNAs were isolated from hyphae collected by filtration with the TRIzol reagent (Invitrogen, Carlsbad, CA, U.S.A.). The first strand cDNA was synthesized with SuperScript™ IV reverse transcriptase (Invitrogen, Carlsbad, CA, USA) and used as the templates for amplification of the *CHE1* fragment containing the PSC with primers CHE1_AMP_F and CHE1_AMP_R (Table S1). The resulting PCR products were purified with the Monarch PCR & DNA Cleanup Kit (New England Biolabs) and sequenced by wide-seq at Purdue Genomic Center.

### Generation of the *che1*, *CHE1*^TAA^, and *CHE1*^TGG^ mutants

To generate the *che1* deletion mutant, the 1,414 bp upstream and 1,268 bp downstream flanking sequences of *CHE1* were amplified with primer pairs CHE1-1F/2R and CHE1-3F/4R (Table S1), respectively. The resulting PCR products were connected to the *hph*-*tk* (the hygromycin phosphotransferase transferase fused with the thymidine kinase) marker cassette (Krappmann et al. 2005) that confers resistance to hygromycin and sensitivity to nucleoside analog floxuridine by overlapping PCR and transformed into protoplasts of strain PH-1 Transformants resistant to hygromycin but sensitive to floxuridine were isolated and verified for deletion of *CHE1* by PCR.

The *CHE1*^TAA^ allele with G^438^ changed to A was generated by overlapping PCR with primer pairs CHE1-7F/CHE1-TAA-R and CHE1-TAA-F/8R (Table S1). After transforming the resulting PCR product into protoplasts of the *che1* mutant, floxuridine-resistant transformants were isolated and confirmed to contain the G^438^ to A mutation by PCR amplification and sequencing (Qi et al. 2024). The same approach was used to generate the *CHE1*^TGG^ mutant with the A^437^ to G mutation with overlapping PCR primers CHE1-TGG-R and CHE1-TGG-F (Table S1).

### Supplementary Information

Supplementary Material 1.

## Data Availability

All data generated or analyzed during this study are included in this published article and its supplementary information files. Experimental materials used in this study are available from the corresponding authors upon request.

## Abbreviations

ADAR: For adenosine deaminase acting on mRNA
ADAT: For adenosine deaminase acting on tRNA
*CHE1*: For conserved hyphal editing 1
IGV: For Integrative Genomics Viewer
PSC: For premature stop codon correction
UTR: For un-translated region

## Declarations

### Data accessibility statement

All the data related to results and conclusion of this manuscript are presented in the main text and supplementary information. Experimental materials used in this study are available from the corresponding authors upon request.
