# Supplementary material for: Hyphal editing of the conserved premature stop codon in CHE1 is stimulated by oxidative stress in Fusarium graminearum
Source: Stress Biol. 2024 Jun 12;4(1):30. doi: 10.1007/s44154-024-00174-w (PMC11169179; doi:10.1007/s44154-024-00174-w)
Supplement: Supplementary file 1 — Supplementary Material 1. [file 44154_2024_174_MOESM1_ESM.pdf]

SUPPLEMENTARY INFORMATION

Hyphal editing of the conserved premature stop codon in *CHE1* is stimulated by oxidative stress in *Fusarium graminearum*

Jingwen Zhou[1†], Yanfei Du[1†], Xiaoxing Xing[1], Panpan Wang[1], Zeyi Wang[2], Huiquan Liu[1], Qinhu Wang[1], JinRong Xu[1,2*]

[1] State Key Laboratory of Crop Stress Biology for Arid Areas and NWAFU-Purdue Joint Research Center, College of Plant Protection, Northwest A&F University, Yangling, Shaanxi 712100, China.

[2] Dept. of Botany and Plant Pathology, Purdue University, West Lafayette, IN 47907. USA.

* Corresponding author:	JinRong Xu
	ORCID#: 0000-0001-5999-5004
	Email: jinrong@purdue.edu
	Tel: 765-496-7496

Table S1. **Primers used in this study**

Fig. S1. Sequence alignment of the Che1 orthologs from closely related *Fusarium* species

The first half (1-145 aa) and second half (147-310 aa) amino acid sequences of Che1 (*Fg*) were aligned with those of its orthologs from *Ft*, *F. tricinctum*; *Fpo*, *F. poae*; *Ffu*, *F. fujikuroi*; *Ffl*, *F. flagelliforme*; *Fgl*, *F. globosum*; *Fc*, *F. circinatum*, the *CHE1* homologs was predicted to encode a 145-aa protein, with the PSC site as the stop codon. Identical and similar amino acid residues are shaded in red and yellow, respectively. Overall, the first half of Che1 (1-145 aa) is less conserved than its second half (147-310 aa) among its orthologs.

Fig. S2. PCR verification of the *che1* deletion mutants

Genomic DNAs from the wild-type strain PH-1 (WT) and three *che1* deletion mutants (Che1-1; 1-25, and 1-27) were used as the templates for amplification with marked primer pairs for lanes 1-4 (Table S1). Primers F7 and R8 are the anchor primers outside the flanking sequences of *CHE1* used for gene replacement (Fig. 4A). Amplifications with primer pairs 7F/H856R and H855F/8R indicate the occurrence of homologous recombination in the upstream and downstream flanking sequences, respectively.

Fig. S3. Verification of the *CHE1*^{TAA} and *CHE1*^{TGG} mutants by PCR and sequencing

A) Genomic DNAs from the wild-type strain PH-1 (WT), *CHE1*^{TAA} mutants (Che1^{TAA}-3 and-4) and *CHE1*^{TGG} (Che1^{TGG}-1, 3, and-4) were used as the templates for amplification with marked

primer pairs for lanes 1-3 (Fig. 4A; Table S1). **B)** PCR products amplified from Che1^{TAA}-3 and Che1^{TGG}-1 with primer pairs FG3G21220-F and FG3G21220-R (Table S1) were sequenced by Sanger sequencing.
